# Supplementary material for: Decreasing the Impact of Anxiety on Cancer Prevention through Online Intervention
Source: Int J Environ Res Public Health. 2020 Feb 5;17(3):985. doi: 10.3390/ijerph17030985 (PMC7038157; doi:10.3390/ijerph17030985)
Supplement: Supplementary file 1 [file ijerph-17-00985-s001.pdf]

Table S1. Differences in level of anxiety associated with selected diseases: baseline and final assessment in intervention and control groups.

		Intervention			Control		
**Disease**	**Level**	**Baseline**	**Final**	p^{**}	**Baseline**	**Final**	p^{**}
**Myocardial infarction**	1	26 (12.6%)	21 (10.1%)		30 (11.7%)	27 (10.5%)	
	2	27 (13.0%)	33 (15.9%)		38 (14.8%)	48 (18.8%)	
	3	46 (22.2%)	56 (27.1%)	0.3	76 (29.7%)	78 (30.5%)	0.2
	4	55 (26.6%)	55 (26.6%)		61 (23.8%)	61 (23.8%)	
	5	53 (25.6%)	42 (20.3%)		51 (19.9%)	42 (16.4%)	
**Brain stroke**	1	14 (6.8%)	13 (6.3%)		17 (6.6%)	15 (5.9%)	
	2	25 (12.1%)	21 (10.1%)		20 (7.8%)	25 (9.8%)	
	3	27 (13.0%)	43 (20.8%)	0.3	47 (18.4%)	52 (20.3%)	0.1
	4	50 (24.2%)	51 (24.6%)		64 (25.0%)	76 (29.7%)	
	5	91 (44.0%)	79 (38.2%)		108 (42.2%)	88 (34.4%)	
**Diabetes**	1	48 (23.2%)	39 (18.8%)		60 (23.4%)	50 (19.5%)	
	2	63 (30.4%)	67 (32.4%)		63 (24.6%)	78 (30.5%)	
	3	62 (30.0%)	69 (33.3%)	0.5	74 (28.9%)	84 (32.8%)	0.4
	4	25 (12.1%)	23 (11.1%)		39 (15.2%)	29 (11.3%)	
	5	9 (4.3%)	9 (4.3%)		20 (7.8%)	15 (5.9%)	
**Cancer**	1	15 (7.2%)	11 (5.3%)		9 (3.5%)	10 (3.9%)	
	2	12 (5.8%)	16 (7.7%)		9 (3.5%)	11 (4.3%)	
	3	23 (11.1%)	34 (16.4%)	0.03	34 (13.3%)	26 (10.2%)	0.6
	4	41 (19.8%)	48 (23.2%)		53 (20.7%)	67 (26.2%)	
	5	116 (56.0%)	98 (47.3%)		151 (59.0%)	142 (55.5%)	
**Asthma**	1	71 (34.3%)	65 (31.4%)		74 (28.9%)	67 (26.2%)	
	2	53 (25.6%)	68 (32.9%)		78 (30.5%)	88 (34.4%)	
	3	52 (25.1%)	50 (24.2%)	0.3	67 (26.2%)	69 (27.0%)	0.9
	4	20 (9.7%)	15 (7.2%)		29 (11.3%)	26 (10.2%)	
	5	11 (5.3%)	9 (4.3%)		8 (3.1%)	6 (2.3%)	
**Flu**	1	98 (47.3%)	105 (50.7%)		142 (55.5%)	133 (52.0%)	
	2	54 (26.1%)	61 (29.5%)		79 (30.9%)	92 (35.9%)	
	3	40 (19.3%)	29 (14.0%)	0.05	28 (10.9%)	23 (9.0%)	0.6
	4	6 (2.9%)	10 (4.8%)		6 (2.3%)	6 (2.3%)	
	5	9 (4.3%)	2 (1.0%)		1 (0.4%)	2 (0.8%)	
**Liver cirrhosis**	1	40 (19.3%)	35 (16.9%)		50 (19.5%)	49 (19.1%)	
	2	32 (15.5%)	40 (19.3%)		43 (16.8%)	50 (19.5%)	
	3	46 (22.2%)	56 (27.1%)	0.1	65 (25.4%)	69 (27.0%)	0.2
	4	48 (23.2%)	46 (22.2%)		55 (21.5%)	55 (21.5%)	
	5	41 (19.8%)	30 (14.5%)		43 (16.8%)	33 (12.9%)	
**Schizophrenia**	1	36 (17.4%)	30 (14.5%)		49 (19.1%)	34 (13.3%)	
	2	15 (7.2%)	30 (14.5%)		24 (9.4%)	35 (13.7%)	
	3	40 (19.3%)	36 (17.4%)	0.2	41 (16.0%)	45 (17.6%)	0.7
	4	43 (20.8%)	52 (25.1%)		45 (17.6%)	53 (20.7%)	
	5	73 (35.3%)	59 (28.5%)		97 (37.9%)	89 (34.8%)	
**AIDS (Acquired Immune Deficiency Syndrome**	1	35 (16.9%)	36 (17.4%)		52 (20.3%)	36 (14.1%)	
	2	18 (8.7%)	26 (12.6%)		25 (9.8%)	35 (13.7%)	
	3	41 (19.8%)	34 (16.4%)	0.4	39 (15.2%)	42 (16.4%)	0.5
	4	37 (17.9%)	39 (18.8%)		48 (18.8%)	56 (21.9%)	
	5	76 (36.7%)	72 (34.8%)		92 (35.9%)	87 (34.0%)	
**TB (Tuberculosis)**	1	41 (19.8%)	42 (20.3%)		30 (11.7%)	47 (18.4%)	
	2	43 (20.8%)	43 (20.8%)		38 (14.8%)	54 (21.1%)	
	3	45 (21.7%)	58 (28.0%)	0.2	76 (29.7%)	68 (26.6%)	<0.001
	4	46 (22.2%)	39 (18.8%)		61 (23.8%)	56 (21.9%)	
	5	32 (15.5%)	25 (12.1%)		51 (19.9%)	31 (12.1%)	

[*] 1 expresses the lowest and 5 highest level of anxiety. [**] statistical significance in Wilcoxon test for paired variables

Commented [m1]: * is not mentioned in the table. Please mention or delete notes for * .
